# Hepatitis C Virus in Blood Donors, Brazil

**DOI:** 10.3201/eid1504.081288

**Published:** 2009-04

**Authors:** Kátia Luz Torres, Adriana Malheiro, Adriana Tateno, Tatiane Amabile de Lima, Laura Patricia Viana Maia, João Paulo Diniz Pimentel, Márcia Poinho Encarnação de Morais, Christiane Santana de Melo Usui, Flavia de Oliveira Braga, Igor Araújo Ferreira Silva, Felicien Vasquez, José Eduardo Levi

**Affiliations:** Fundação de Hematologia e Hemoterapia do Amazonas, Manaus, Amazon, Brazil (K.L. Torres, A. Malheiro, T.A. de Lima, L.P.V. Maia, J.P.D. Pimentel, M.P.E. de Morais, C.S. de Melo Usui, F. Vasquez); Universidade Federal do Amazonas, Manaus (A. Malheiro, F. de Oliveira Braga, I.A.F. Silva); Instituto de Medicina Tropical de São Paulo, São Paulo, Brazil (J.E. Levi)

**Keywords:** HCV, blood donors, Amazon, hepatitis C virus, molecular characterization, serologic characterization, immunoblot, polymerase chain reaction, algorithm, letter

**To the Editor:** The Fundação de Hematologia e Hemoterapia do Amazonas is a public health service in Manaus, Brazil, that is responsible for serologic screening of all blood donations in the region. In the state of Amazon, 9.0% of donated blood is discarded on the basis of serologic findings; discarding because of hepatitis C virus (HCV) antibodies declined from 1.25% in 1995 to 0.32% in 2007. The aim of this study was to characterize the serologic and molecular profile of HCV-antibody–positive blood donors from the Fundação de Hematologia e Hemoterapia do Amazonas.

For the study, 154 donors were selected from a routine database of voluntary blood donors who had donated from September 2005 through April 2007 (82,851 donations). Fresh plasma samples were sent to the laboratory in Manaus through the usual transportation systems for regular donations; i.e., samples from 27 cities are transported by air for ≈2 hours, and samples from 21 localities are transported by boat or road, all under refrigerated conditions.

An in-house standardized nested-PCR was used to detect HCV RNA (*1*). Genotype assignment was based on type-specific motifs on the sequenced amplicons delimited by primers HC11/HC18 from the 5′ untranslated region (*2*). Viral load was determined by commercial assay (HCV Monitor, Roche Molecular Systems, Inc., Branchburg, NJ, USA).

 An association was observed between HCV RNA and donor age; the same trend was seen in the first-time blood donor group. Associations between HCV-RNA detection and gender (p = 0.875) and place of donation (p = 0.989) were not significant. Using 18–25 years of age as the reference group, we found that odds ratios (ORs) for having HCV viremia were higher for those 45–55 years of age (OR 8.19, p<0.001) and 35–45 years of age (OR 3.49, p = 0.003).

We observed increasing rates of RNA detection according to the signal-to-cutoff (S/CO) ratio. However, some donors had a weak S/CO ratio (between 1 and 2) with positive nested-PCR tests (Figure). Although adopting an S/CO ratio as a criterion for referring for further testing by recombinant immunoblot assay (RIBA) has been advocated by some groups (*3*), our data show that this criterion may be misleading and would deny a confirmatory diagnosis by giving false-negative results for many persons.

**Figure:**
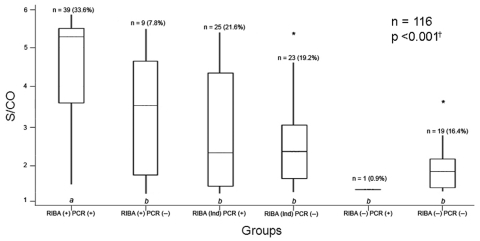
Distribution of hepatitis C virus (HCV) enzyme immunoassay signal-to-cutoff (S/CO) values by recombinant immunoblot assay (RIBA) interpretations among HCV-RNA–positive [PCR (+)] and HCV-RNA–negative [PCR (−)] donated blood samples. Group *a* differs statistically from all groups *b* with 95% confidence intervals. The Mann-Whitney test was used to compare the 2 groups. (+), positive; (−), negative; (Ind), indeterminate. *S/CO values outside interquartile intervals; †Kruskal-Wallis test.

A total of 113 samples were analyzed by RIBA; among 48 RIBA-reactive samples, 9 (18.8%) were negative for HCV RNA in plasma. However, because PCR results may sometimes be negative for persons who are actually infected, a single negative PCR result should not be relied on as evidence that virus has cleared from plasma. Such patients must be observed for years before they may be declared cured (*4*).

Among 97 RIBA-positive or -indeterminate samples, viral load was detectable in only 33 samples: 27 (81.8%) RIBA-positive samples and 6 (18.2%) RIBA-indeterminate samples. Only HCV genotypes 1 (87.1%) and 3 (12.9%) were found. Geographic distribution shows genotypes 1 and 3 in Manaus and only genotype 1 in other Amazon cities. This genotype geographic distribution is similar to that found for many Brazilian cities and Eastern countries and may reflect the route of HCV introduction into the Amazon; the virus was probably brought to the Amazon region by European immigrants and blood-derived medicines imported to Brazil. This hypothesis is corroborated by the finding of genotype 3 exclusively in Manaus, suggesting that this city is the point of arrival of HCV and that new strains were disseminated from Manaus to inner localities. Historical reconstruction of HCV in Amazon could be attempted by using these isolates as well as others from hepatitis patients in the region, including genotype 2 (*5*).

We found a higher-than-expected rate of 50% for indeterminate immunoblot results among samples that were HCV-RNA positive by nested PCR. The presence of HCV RNA in plasma samples from 70%–75% of blood donors with indeterminate immunoblot results has also been reported by other groups in Brazil (*6**,**7*); however, in contrast, other investigators have reported RNA prevalence in such samples to be ≈2.5% (*1*,*8*). Indeterminate RIBA test results can indicate seroconversion or seroreversion or, occasionally, a chronic infection when RNA HCV is detected in plasma (*9**,**10*). To provide better understanding of the meaning of these indeterminate results, ongoing follow-up studies are examining the immune status of these persons.

Our data offer insights for counseling of donors who have repeatedly HCV-reactive results. We suggest that Amazon region blood banks screen by enzyme immunoassay and use molecular testing as the first supplemental test and that immunoblot be applied to the remaining HCV-RNA nonreactive samples to distinguish between true and false anti-HCV carriers. This new algorithm would save considerable resources currently spent on immunoblot-indeterminate persons in addition to HCV-RNA reactive persons who do not require further testing for confirmation. Moreover, according to current policy, those with false-positive results may be reinstated as donors if they have negative retesting results after 6 months.
